# Depletion of protein kinase C (PKC) by 12- *O* -tetradecanoylphorbol-13-acetate (TPA) enhances platinum drug sensitivity in human ovarian carcinoma cells

**DOI:** 10.1054/bjoc.1999.0873

**Published:** 1999-12-08

**Authors:** S Isonishi, K Ohkawa, T Tanaka, S B Howell

**Affiliations:** Departments of Obstetrics/Gynecology Biochemistry, Jikei University School of Medicine, 3-25-8 Nishi-shinbashi, Minato-ku, 105, Japan; The Cancer Center, University of California, San Diego, La Jolla, 92093, USA

**Keywords:** platinum agents, protein kinase C, drug sensitivity

## Abstract

Down-regulation of protein kinase C (PKC) by 12- *O* -tetradecanoylphorbol-13-acetate (TPA) enhances the sensitivity of human ovarian carcinoma 2008 cells to various types of platinum compounds such as cisplatin (DDP), carboplatin and (–)-(R)-2-aminomethylpyrrolidine (1,1-cyclobutanedicarboxylato)-platinum(II) monohydrate (DWA) by a factor of two- to threefold. TPA enhanced the sensitivity of the DDP-resistant 2008/C13*5.25 subline to each of these three drugs to the same extent as for the 2008 cells. The extent of PKC down-regulation and drug sensitization depended on the duration of TPA exposure; maximum effect was achieved with a 48 h pretreatment. Sensitization was TPA concentration-dependent and was maximal at 0.05 μM TPA. 2008 cells expressed only the PKCα and PKCζ isoforms. Western blot analysis revealed that whereas the expression of PKCα was reduced by TPA the level of PKCζ was not affected. These results suggest that PKCα is the isotype responsive to TPA in these cells and that platinum drug sensitivity can be modulated by this isoform alone. In parallel to its effect on PKCα, TPA decreased cellular glutathione content by 30 ± 3 (standard deviation (s.d.) % in 2008 cells and by 41 ± 3 (s.d.) % in 2008/C13*5.25 cells. TPA also increased accumulation of DDP and DWA by 70%, although this effect was limited to the 2008/C13*5.25 cells. TPA rendered 2008 and 2008/C13*5.25 cells resistant to cadmium chloride by a factor of 3.7 and 3.6-fold respectively, suggesting a significant increase in cellular metallothionein content. Although the mechanism of TPA induced sensitization is not yet fully understood, this study points to a central role for PKCα in modulating platinum drug sensitivity. © 2000 Cancer Research Campaign


*British Journal of Cancer* (2000) **82**(1), 34–38
© 2000 Cancer Research Campaign
Article no. bjoc.1999.0873

### Depletion of protein kinase C (PKC) by 12-*O*-tetradecanoylphorbol-13-acetate (TPA) enhances platinum drug sensitivity in human ovarian carcinoma cells

**S Isonishi[1], K Ohkawa[2], T Tanaka[1] and SB Howell[3]**

Departments of [1]Obstetrics/Gynecology and [2]Biochemistry, Jikei University School of Medicine, 3-25-8 Nishi-shinbashi, Minato-ku, Tokyo 105, Japan; [3]The Cancer Center, University of California, San Diego, La Jolla, CA 92093, USA

**Summary** Down-regulation of protein kinase C (PKC) by 12-*O*-tetradecanoylphorbol-13-acetate (TPA) enhances the sensitivity of human ovarian carcinoma 2008 cells to various types of platinum compounds such as cisplatin (DDP), carboplatin and (–)-(R)-2-aminomethylpyrrolidine (1,1-cyclobutanedicarboxylato)-platinum(II) monohydrate (DWA) by a factor of two- to threefold. TPA enhanced the sensitivity of the DDP-resistant 2008/C13*5.25 subline to each of these three drugs to the same extent as for the 2008 cells. The extent of PKC down-regulation and drug sensitization depended on the duration of TPA exposure; maximum effect was achieved with a 48 h pretreatment. Sensitization was TPA concentration-dependent and was maximal at 0.05 μM TPA. 2008 cells expressed only the PKCα and PKCζ isoforms. Western blot analysis revealed that whereas the expression of PKCα was reduced by TPA the level of PKCζ was not affected. These results suggest that PKCα is the isotype responsive to TPA in these cells and that platinum drug sensitivity can be modulated by this isoform alone. In parallel to its effect on PKCα, TPA decreased cellular glutathione content by 30 ± 3 (standard deviation (s.d.) % in 2008 cells and by 41 ± 3 (s.d.) % in 2008/C13*5.25 cells. TPA also increased accumulation of DDP and DWA by 70%, although this effect was limited to the 2008/C13*5.25 cells. TPA rendered 2008 and 2008/C13*5.25 cells resistant to cadmium chloride by a factor of 3.7 and 3.6-fold respectively, suggesting a significant increase in cellular metallothionein content. Although the mechanism of TPA induced sensitization is not yet fully understood, this study points to a central role for PKCα in modulating platinum drug sensitivity. © 2000 Cancer Research Campaign

**Keywords**: platinum agents; protein kinase C; drug sensitivity

Cisplatin (DDP) is active against several types of human cancer, particularly those of the ovary, testis, bladder and the head and neck (Loehrer and Einhorn, 1984). However, its efficacy is limited by tumour cell resistance, present either at the onset of treatment or evolving after an initial treatment response (Ozols and Young, 1984). Studies using isogenic pairs of sensitive and resistant cells have shown that acquired DDP resistance is mediated by multiple mechanisms including reduced intracellular accumulation, elevated intracellular thiol content and increased DNA repair of platinum-induced inter- or intrastrand DNA cross-links (Andrews and Howell, 1990). There is substantial interest in developing pharmacological strategies for overcoming acquired DDP resistance by modulating these parameters (Schilder and Ozols, 1992); however, at the present time the only way to achieve this is to administer larger doses of the drug.

Participation of protein kinase C (PKC) in intracellular signalling has been demonstrated in many cell types, including variety of cancer cells (Hsu et al, 1998). PKC-mediated phosphorylation of numerous protein substrates is associated with a wide range of biological effects, including induction of cellular proliferation and differentiation, activation of nuclear transcription factors and cell surface receptors, and tumour promotion (Craven and DeRubertis, 1988; Rahmsdorf et al, 1990; Brach et al, 1992). PKC is a family of at least nine structurally related serine/threonine kinase isoforms differing in substrate specificity and dependence on $Ca^{2+}$ availability. Differential regulation of the activation of different forms of PKC is not well understood. Calcium concentrations are probably important, as one group of isozymes (α, βI, βII and γ) are regulated by $Ca^{2+}$, phosphatidylserine and diacylglycerol (DAG). However, the activities of the more recently discovered isozymes (ε, δ, η, and θ) are independent of $Ca^{2+}$, and ζ is independent of both $Ca^{2+}$ and DAG. PKC isozyme function can be studied using antisense technology. Balboa et al (1994) selectively reduced the levels of either PKCα or PKCβI by transfection of kidney D1 cells with corresponding antisense oligonucleotides. This study implicated PKCα but not PKCβI in the activation of phospholipase C.

PKC has been identified as a high-affinity receptor for the phorbol ester TPA (Niedel et al, 1983). 12-*O*-tetradecanoylphorbol-13-acetate (TPA) is one of a group of tumour promoters which can either stimulate cell proliferation or cause arrest, depending on the type of cell which is treated and proliferative status of the culture. Through activation of PKC, treatment of cells with TPA can lead to a number of changes in phenotype as a result

*Received 9 February 1999*
*Revised 21 June 1999*
*Accepted 16 July 1999*

*Correspondence to:* S Isonishi of PKC-dependent phosphorylation, including alteration of cellular sensitivity to platinum drugs (Isonishi et al, 1990). Our previous studies showed that activation of PKC by TPA was able to circumvent acquired DDP resistance by enhancing sensitivity to the clinically utilized platinum drugs, with the exception of (–)-(R)-2-aminomethylpyrrolidine (1,1-cyclobutanedicarboxylato)-platinum (II) monohydrate (DWA), in human ovarian carcinoma cells (Isonishi et al, 1994*a*, 1994*b*). However, it is not known which isoforms mediate this effect. The aim of the current study was to investigate the effect of selective down-regulation of the PKCα isozyme on platinum drug sensitivity.

### MATERIALS

DDP and carboplatin (CBDCA) were obtained from the Bristol-Myers Squibb K.K., Japan. DWA was obtained from the Chugai Pharmaceutical Co. TPA and cadmium chloride ($CdCl_2$), leupeptin, and phenylmethanesulphonyl fluoride (PMSF) were purchased from Sigma Chemical Co. (Tokyo, Japan). Monoclonal anti-PKC antibodies were purchased from Seikagaku Co., Tokyo, Japan.

### METHODS

### Tumour cell lines

The human cell line 2008 was established from a patient with a serous cystadenocarcinoma of the ovary (Disaida et al, 1972). A resistant subline, designated 2008/C13*5.25, was obtained by 13 monthly selections with 1 μM DDP (Andrews et al, 1985). The cells were grown on tissue culture dishes in a humidified incubator at 37°C and 5% carbon dioxide atmosphere.

### TPA treatment and colony assays

Colony forming assays were used to assess the effect of TPA on the sensitivity of each drug. Five millilitres of cell suspension, containing 600 cells, were plated on 60-mm polystyrene tissue culture dishes (Corning Glass Works, Corning, NY, USA). Drug solution was added to triplicate plates at each drug concentration. After a 48 h pre-incubation in the presence or absence of 0.1 μM TPA followed by 1 h exposure to platinum with or without TPA, the drug-containing medium was aspirated and replaced with drug-free medium. After 10 days colonies of over 60 cells were counted macroscopically.

### Calculation of $IC_{50}$ values and enhancement factors

$IC_{50}$ was defined as the drug concentration reducing the number of colonies by 50% and was determined by linear regression analysis of the data. The change in drug sensitivity was expressed as the ratio of the $IC_{50}$ values for the control and TPA-treated cells.

### Platinum accumulation

Subconfluent monolayers were treated with 37°C RPMI-1640 medium containing 120 μM DDP or DWA. After a 1 h exposure, the cells were washed rapidly with 4°C phosphate-buffered saline (PBS) four times. Two millilitres of 1 N sodium hydroxide was added and the cells were allowed to digest. A 20 μl aliquot was used for determination of protein content by the method of Bradford (1976), and the remaining was analysed in an atomic absorption spectrometer (Hitachi, Z 8000).

### Preparation of cell lysates and subcellular fractions

Subconfluent 2008 and 2008/C13*5.25 cells grown in 150-mm tissue culture dishes were used to prepare cell lysates for determination of PKC content. After incubation for 48 h in the presence or absence of 0.1 μM TPA, monolayers of cells were rapidly rinsed twice with ice-cold PBS and lysed with buffer solution containing 50 mM Tris–HCl, 5 mM EDTA, 10 mM EGTA, 0.1 mM leupeptin, 0.3% (w/v) mercaptoethanol, and 50 mg $ml^{-1}$ phenylmethanesulphonyl fluoride (PMSF). The solubilized cellular material was harvested by scraping from culture dishes then centrifuged at 12 000 ***g*** for 5 min at 4°C. Supernatant samples were stored at –70°C until used. For the preparation of membrane and cytosolic subcellular fractions the scraped material was sonicated for 30 s at 4°C and then centrifuged at 100 000 ***g*** for 60 min at 4°C. The isolated supernatant sample was designated the cytosolic fraction. The precipitated material was subsequently sonicated and designated the membrane-associated fraction.

### Western blot analysis of PKC

Cell extracts were boiled for 5 min and fractionated using sodium dodecyl sulphate polyacrylamide gel electrophoresis (SDS-PAGE) minigels (7.5% separating gel) followed by electrotransfer to nitrocellulose paper. The blots were incubated with 1 μg $ml^{-1}$ of mouse anti-PKC monoclonal antibodies specific for the α, β, γ, δ, ε, μ and ζ isotypes followed by horseradish peroxidase conjugated anti-mouse Ig (1:4000 dilution).

### Assay of PKC activity

PKC activity was measured using a kit (Amersham, RPN 77A). The reaction was initiated by addition of 25 μl of protein sample to a reaction mixture containing 12 mM calcium acetate, 50 mM Tris–HCl, 0.05% (w/v) sodium azide (pH 7.5), 8 mole% Lα phosphatidyl-L-serine, 900 μM peptide substrate, 150 μM magnesium [$^{32}$P]ATP, 45 mM magnesium acetate, 30 mM dithiothreitol in a total volume of 75 ml. After incubation for 15 min at 25°C, aliquots of the reaction mixture were spotted onto the binding paper squares, and the squares were placed in 75 mM orthophosphoric acid for 10 min. Radioactivity retained on the papers was determined. The $^{32}$P incorporated into the synthetic peptide, quantitatively measured by counting the radioactivity of the binding papers, is a direct measure of PKC activity which was expressed as $^{32}$P incorporated $min^{-1}$ $mg^{-1}$ protein.

### Measurement of GSH content

GSH content was measured as previously reported (Reed et al, 1980). After the monolayers were incubated in the presence or absence of TPA for 48 h, the cell pellets were prepared and incubated in the dark for 15 min. An equal volume of 4 M sodium methane sulphonate was added to each tube, which was then frozen until assayed by high performance liquid chromatography (HPLC).

### RESULTS

*© 2000 Cancer Research Campaign*

**Table 1** Effect of TPA on sensitivity to DDP, CBDCA, and DWA

| Drug | Cell | IC50[1] TPA treatment (–) | IC50[1] TPA treatment 48 hr | Enhancement facter[2] (-fold) |
|---|---|---|---|---|
| DDP | 2008 | 3.1 ± 0.6 | 1.1 ± 0.2 | 2.8 ± 0.6 |
| | C13*5[3] | 13.6 ± 2.7 | 6.3 ± 1.2 | 2.2 ± 0.4 |
| CBDCA | 2008 | 22.6 ± 5.8 | 7.9 ± 0.8 | 2.9 ± 0.7 |
| | C13*5 | 107.4 ± 0.3 | 20.9 ± 5.2 | 5.1 ± 0.0 |
| DWA[4] | 2008 | 118.9 ± 9.8 | 53.7 ± 8.8 | 2.2 ± 0.2 |
| | C13*5 | 42.6 ± 1.8 | 22.5 ± 6.8 | 1.9 ± 0.1 |

[1] 50% Inhibitory concentration; μM, mean ± s.d. [2] Enhancement factor = $IC_{50}$ (control)/$IC_{50}$ (TPA). [3] 2008/C13*5.25. [4] DWA.

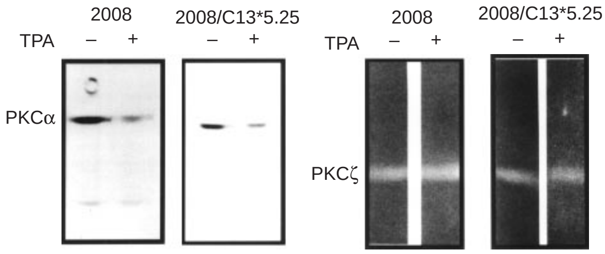

**Figure 1** Effect of TPA on PKC expression. PKC was extracted from 2008 and 2008/C13*5.25 cells, then Western blot analyses were performed using polyclonal antisera specific to PKCα and PKCζ. Densitometric analysis showed that TPA decreased the expression of PKCα to 60.3 ± 4.4% of control in 2008 cells and to 45.0 ± 2.8% (s.d.; $n = 3$) ($P < 0.01$) in 2008/C13*5.25 cells. TPA exposure had no effect on the level of PKCζ

### Effect of TPA on platinum drug sensitivity

The data presented in Table 1 show that 48 h pretreatment of the DDP-sensitive 2008 and DDP-resistant 2008/C13*5.25 cells with 0.1 μM TPA increased cellular sensitivity to DDP by a factor of 2.8 ± 0.5 and 2.2 ± 0.4 (standard deviation (s.d.) $n = 4$) ($P < 0.01$) respectively. TPA also enhanced sensitivity to CBDCA in both cell lines to the same extent as for DDP. The sensitization effect was dependent on TPA concentration and the maximum sensitization effect was achieved with as little as 0.05 μM TPA. TPA was also able to enhance cellular sensitivity to DWA by a factor of 2.2 ± 0.2-fold in 2008 cells and 1.9 ± 0.1-fold in 2008/C13*5.25 cells (s.d.; $n = 4$) ($P < 0.01$).

### PKC isoform analysis

2008 cells express only the α and ζ isoforms of PKC. To determine whether TPA sensitization was related to changes in the level of either of these two isoforms, a Western blot analysis of total cell extracts was carried out using PKC antibodies specific for either PKCα or PKCζ. Figure 1 shows that a 48 h exposure to TPA reduced the PKCα levels in both the 2008 and 2008/C13*5.25 cells. Densitometric analysis of the bands showed that TPA decreased the expression of PKCα to 60.3 ± 4.4% of control in 2008 cells and to 45.0 ± 2.8% (s.d.; $n = 3$) ($P < 0.01$) in 2008/C13*5.25 cells. In contrast, TPA exposure had no effect on the level of PKCζ. It thus appears that, in these two cell lines, there was a differential effect of TPA on these two isoforms.

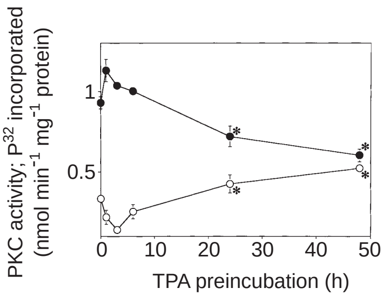

**Figure 2** Time course of PKC activity in 2008 cells. Membrane (solid circles) and cytosol (open circles) associated PKC activity in 2008 cells treated with 0.1 μM TPA for various periods of time. Points, mean values of 3 experiments performed with triplicate cultures; bars, s.d. Asterisks, $P < 0.05$ compared to the control at the same time point

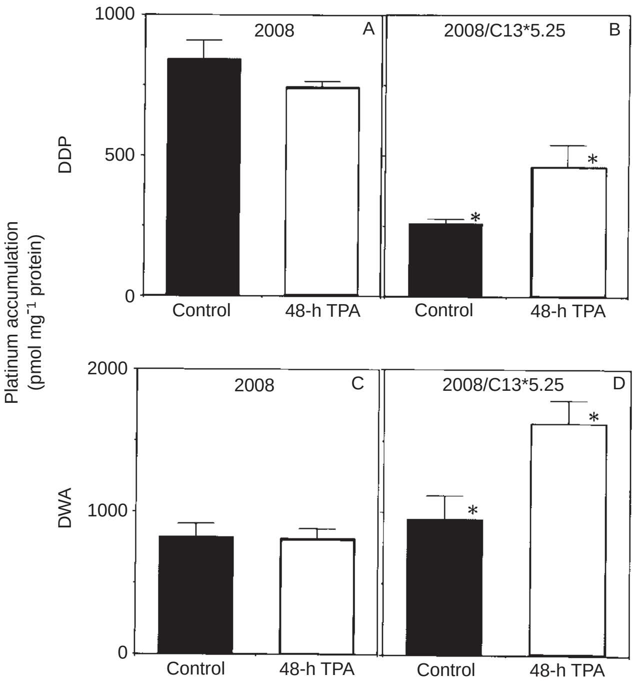

**Figure 3** Effect of TPA on cellular platinum accumulation. Platinum content after a 1 h exposure to either 120 μM DDP or DWA alone (hatched bars) or after a concurrent 1 h exposure to 0.1 μM TPA with either 120 μM DDP or DWA following 48 h pre-incubation in the presence of 0.1 μM TPA (open bars). In 2008/C13*5.25 cells TPA increased DDP accumulation by 1.8 ± 0.3 (s.d.; $n = 4$)-fold and DWA accumulation by 1.7 ± 0.2 (s.d.; $n = 4$)-fold ($P < 0.01$) (B and D)

### Time course of PKC activity

To better characterize the changes in PKC activity found in 2008 cells, the subcellular distribution of PKC activity was determined as a function of time after the start of exposure to 0.1 μM TPA. Figure 2 shows that during the first 6 h there was a rapid loss of PKC activity from the cytosolic fraction. Concomitantly, there was a prompt increase in plasma membrane-associated PKC activity. With continued exposure there was a progressive decrease in the membrane-associated activity, and recovery of the cytosolic activity to above baseline level by 48 h.

### Effect of TPA on cellular platinum accumulation

© 2000 Cancer Research Campaign

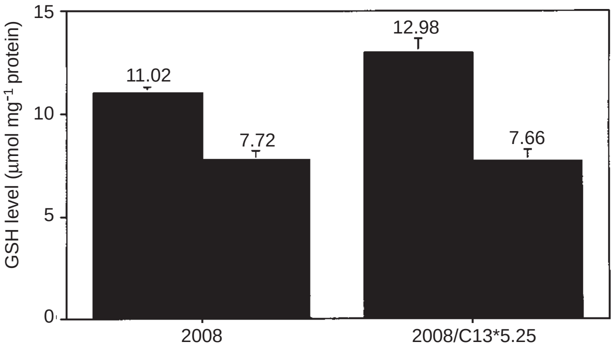

**Figure 4** Effect of TPA on GSH content. Intracellular GSH content in 2008 and 2008/C13*5.25 cells immediately after a 48 h pre-incubation in the presence (solid bars) or absence (hatched bars) of 0.1 µM TPA. Bars, mean of 3 independent experiments, each with duplicate cultures; bars, SD. TPA treatment decreased GSH level by 30 ± 3 (s.d.)% ($P < 0.01$) in 2008 cells and by 41 ± 3 (s.d.)% ($P < 0.01$) in 2008/C13*5.25 cells

Cells were pre-incubated with 0.1 µM TPA for 24 or 48 h and then with either DDP or DWA for 1 h. TPA did not modulate the cellular accumulation of either DDP or DWA in 2008 cells at either time point. However, in the 2008/C13*5.25 cells TPA increased DDP accumulation at both time points to the same degree by 1.8 ± 0.3 (s.d.; $n = 4$)-fold and DWA accumulation by 1.7 ± 0.2 (s.d.; $n = 4$)-fold (Figure 3, B, D) ($P < 0.01$ for both). Thus, TPA-mediated sensitization was associated with an increase in drug accumulation in the DDP-resistant 2008/C13*5.25 cells but not the DDP-sensitive 2008 cells.

### Effect of TPA on cellular GSH and MT content

Changes in GSH and MT levels are among several other mechanisms that have been reported to modulate DDP sensitivity. Figure 4 shows the cellular GSH content measured by HPLC analysis. TPA treatment significantly decreased GSH level by 30 ± 3 (s.d.)% in the 2008 cells and by 41 ± 3 (s.d.)% in 2008/C13*5.25 cells, an effect consistent with enhanced sensitivity to DDP. In contrast, TPA rendered 2008 and 2008/C13*5.25 cells resistant to $CdCl_2$ by 3.7 ± 1.1 (s.d.)-fold and 3.6 ± 0.7 (s.d.)-fold, suggesting a substantial increase in cellular MT content, an effect that would be expected to reduce rather than increase sensitivity to DDP.

### DISCUSSION

Several lines of evidence indicate that modulation of PKC activity can alter cellular sensitivity to cytotoxic agents. Results of cytotoxicity studies in which PKC activity was down-regulated with either TPA or the inhibitor H-7 (1-5-isoquinolinesulphonyl)-2-methylpiperazine) indicate a close relationship between PKC activity and cellular sensitivity to several classes of anticancer drugs (Fine et al, 1988; Basu et al, 1990; Isonishi et al, 1994). P-glycoprotein, which mediates resistance to many chemotherapeutic drugs, has been reported to be a good substrate for PKC (Chambers et al, 1990), and enhanced phosphorylation of this protein has been noted following phorbol ester treatment. However the cells we have used in this experiments did not express P-glycoprotein at detectable levels as determined by Western blot (data not shown). The fact that sensitivity to several different classes of drugs with different cytotoxic mechanisms is affected suggests that PKC works through several different pathways to alter drug sensitivity.

One of the major findings of the current study is that TPA selectively down-regulated the level of PKCα but not that of PKCζ in 2008 cells. This documents a differential effect of TPA on at least these two isoforms, and directs attention to PKCα as a candidate mediator of the TPA effect on DDP sensitivity. A recent report using antisense cDNA against PKCα and PKCβ1 has demonstrated that only PKCα mediates the phorbol ester activation of phospholipase D in Madin-Darby canine kidney D1 cells (Balboa et al, 1994). This is consistent with our observation that PKCα was responsive to TPA treatment in the 2008 cells. Total membrane associated PKC activity was not completely down-regulated even after a 48 h exposure to TPA probably because PKCζ activity was not altered by this treatment. As yet it is not possible to selectively measure just PKCα activity. Such a measurement would enable one to directly test whether the relatively small changes observed in the level of PKCα reflect a change in actually commensurate with the change in sensitivity to DDP.

The cell line used in this study expressed a limited number of PKC isozymes. As has been reported previously (Isonishi et al, 1994) electrophoretic analysis with antisera specific for the α, β, γ, δ, ε, µ and ζ isotypes of PKC indicated that α- and ζ-isotype PKC (PKCα and ζ) were the dominant forms present in both 2008 and 2008/C13*5.25 cells, whereas none of the other isoforms could be identified in these cells. Cell lines that express other specific PKC isozymes should be tested to determine whether other isoforms are involved in regulating platinum drug sensitivity. One of the major challenges in identifying exactly how TPA modulates drug sensitivity is the fact that PKC has multiple substrates, and is involved in several different signalling pathways (Rapp et al, 1991; Bruder et al, 1992; Kolch et al, 1993; Chmura et al, 1966; Blagosklonny et al, 1997) through its ability to initiate phosphorylation cascades (McCaffrey et al, 1987; Smeal et al, 1992; Fung et al, 1997). Sorting out which isoforms are modulated by TPA, and which can mediate enhanced DDP sensitivity, should permit identification of the most important signal transduction pathways involved.

Our studies also indicate that the effect of TPA varies with the underlying DDP-sensitivity of the cell. While TPA enhanced the DDP sensitivity to almost the same degree in the 2008 and 2008/C13*5.25 cells, the mechanisms by which it accomplished this were not the same. TPA increased DDP uptake in the resistant but not in the sensitive cells. This is of particular interest because impaired uptake of DDP is one of the major mechanisms of DDP resistance in the 2008/C13*5.25 cells. It has been postulated that DDP accumulation is partly due to passive diffusion and partly due to facilitated diffusion through a gated channel, and that reduced DDP accumulation in resistant cells may result from inactivation of a channel protein (Gately, 1993). The fact that TPA can modulate DDP uptake provides another piece of evidence indicating that uptake is regulatable, and supports the hypothesis that the signal transduction pathway activated by TPA in the 2008/C13*5.25 cells links to the molecular mechanism that regulates DDP accumulation.

Elevated levels of GSH, the major intracellular non-protein thiol, have been observed in human ovarian carcinoma cells with acquired DDP-resistance (Godwin, 1992). Likewise, on the basis of experiments in mice in which the gene has been knocked out, the level of MT, the major intracellular protein thiol, has been reported to play an important role in controlling DDP sensitivity (Kondo, 1995). Over-expression of MT has been observed in a number of human tumour cell lines with acquired DDP-resistance

© 2000 Cancer Research Campaign 

(Kelly, 1988). In our experiments, TPA treatment produced a substantial decrease in GSH level, but changes in $CdCl_2$ sensitivity indicative of an increase in MT level. It is hard to know whether either of these effects participated in changing DDP sensitivity, or whether they just neutralized each other. Likewise, it is not clear whether either effect results from a direct biochemical action of TPA or an indirect effect mediated via activation of PKC. Although we did not measure the effect of TPA on platinum-DNA adduct formation, based on prior studies an 80% increase in platinum accumulation combined with a 40% decrease in GSH content would not appear to be of sufficient magnitude to account for two- to fivefold increase in sensitivity to the various platinum drugs tested. This lack of correlation is likely due to the involvement of multiple mechanisms in the regulation of platinum drug sensitivity.

### ACKNOWLEDGEMENTS

The authors wish to thank Doreen K Hom, Cancer Center, University of California, San Diego, for her advice and assistance.

### REFERENCES

Andrews PA and Howell SB (1990) Cellular pharmacology of cisplatin: perspectives on mechanisms of acquired resistance. *Cancer Cells* **2**: 35–43

Andrews PA, Murphy MP and Howell SB (1985) Differential potentiation of alkylating and platinating agent cytotoxicity in human ovarian carcinoma cells by glutathione depletion. *Cancer Res* **45**: 6250–6253

Balboa MA, Firestein BL, Godson C, Bell KS and Insel PA (1994) Protein kinase C alpha mediates phospholipase D activation by nucleotides and phorbol ester in Madin-Darby canine kidney cells. Stimulation of phospholipase D is independent of activation of polyphosphoinositide-specific phospholipase C and phospholipase A2. *J Biol Chem* **269**: 10511–10516

Basu A, Teicher BA and Lazo JS (1990) Involvement of protein kinase C in phorbor ester-induced sensitization of Hela cells to cis-diamminedichloroplatinum(II). *J Biol Chem* **265**: 8451–8457

Blagosklonny MV, Prabhu NS and El-Deiry WS (1997) Defects in P21$^{WAF1/CIP1}$, Rb, and c-myc signaling in phorbol-resistant cancer cells. *Cancer Res* **57**: 320–325

Brach MA, Herrmann F and Kufe DW (1992) Activation of the AP-1 transcription factor by arabinofuranosylcytosine in myeloid leukemia cells. *Blood* **79**: 728–734

Bradford MM (1976) A rapid and sensitive method for the quantitation of microgram quantities of protein using the principal of protein-dye binding. *Anal Biochem* **72**: 248–254

Bruder JT, Heidecker G and Rupp UR (1992) Serum-, TPA-, and Ras-induced expression from Ap-1/Ets-driven promoters requires Raf-1 kinase. *Genes and Dev* **6**: 545–556

Chambers TC, McAvoy EM, Jacobs JW and Eilon G (1990) Protein kinase C phosphorylates P-glycoprotein in multidrug resistant human KB carcinoma cells. *J Biol Chem* **265**: 7679–7687

Chmura SJ, Nodzenski E, Weichselbaum II, RR and Quintans J (1966) Protein kinase C inhibition induces apoptosis and ceramide production through activation of a neutral sphingomyelinasel. *Cancer Res* **56**: 2711–2714

Craven PA and DeRubertis D (1988) Role of activation of protein kinase C in the stimulation of colonic epithelial proliferation by unsatulated fatty acids. *Gastroenterology* **95**: 676–685

Disaida PJ, Sinkovics JG, Rutledge FN and Smith JP (1972) Cell-mediated immunity to human malignant cells. *Am J Obstet Gynecol* **114**: 979–989

Fine RL, Patel J and Chabner BA (1988) Phorbol esters induce multidrug resistance in human breast cancer cells. *Proc Natl Acad Sci USA* **85**: 582–586

Fung H, Quinlan TR, Janssen YMW, Timblin CR, Marsh JP, Heintz NH, Taatjes DJ, Vacek P, Jaken S and MossmanII BT (1997) Inhibition of protein kinase C prevents asbestos-induced c-fos and c-jun proto-oncogene expression in mesothelial cells. *Cancer Res* **57**: 3101–3105

Gately DP and Howell SB (1993) Cellular accumulation of the anticancer agent cisplatin: a review. *Br J Cancer* **67**: 1171–1176

Godwin AK, Meister A, O'Dwyer PJ, Huang CS, Hamilton TC and Anderson ME (1992) High resistance to cisplatin in human ovarian cancer cell lines is associated with marked increase of glutathione synthesis. *Proc Natl Acad Sci* **89**: 3070–3074

Hsu SL, Chou YH, Yin SC and Liu JY (1998) Differential effects of phorbol ester on growth and protein kinase C isoenzyme regulation in human hepatoma Hep3B cells. *Biochem J* **33**: 57–64

Isonishi S, Andrews PA and Howell SB (1990) Increased sensitivity to cis-diamminedichloroplatinum(II) in human ovarian carcinoma cells in response to treatment with 12-*O*-tetradecanoylphorbol 13-acetate. *J Biol Chem* **265**: 3623–3627

Isonishi S, Hom DK, Eastman A and Howell SB (1994*a*) Enhancement of sensitivity to platinum(II)-containing drugs by 12-*O*-tetradecanoylphorbol-13-acetate in a human ovarian carcinoma cell line. *Br J Cancer* **69**: 217–221

Isonishi S, Ochiai K, Yasuda M, Ohkawa K and Terashima Y (1994*b*) Mechanism-related circumvention of cisplatin resistance in human ovarian carcinoma cells by (–)-(R)-2-aminomethylpyrrolidine (1,1-cyclobutanedicarboxylato)-platinum(II) monohydrate and modulation of its sensitivity by 12-*O*-tetradecanoylphorbol-13-acetate. *Int J Oncol* **5**: 1309–1314

Kelly SL, Basu A, Teicher BA, Hacker MP, Hamer DH and Lzo JS (1988) Overexpression of metallothionein confers resistance to anticancer drugs. *Science* (Washington DC), **241**: 1813–1815

Kolch W, Heldecker G, Kochs G, Hummel R, Vahida H, Mischak H, Finkenzeller G, Marmé D and Rapp UR (1993) Protein kinase C$\alpha$ activates raf-1 by direct phosphorylation. *Nature* **364**: 249–252

Kondo Y, Woo ES, Michalska AE, Andy Choo KH and Lazo J (1995) Metallothionein null cells have increased sensitivity to anticancer drugs. *Cancer Res* **55**: 2021–2023

Loehrer PJ and Einhorn LH (1984) Cisplatin. *Ann Inter Med* **100**: 704–713

McCaffrey R, Ran W, Camisi J and Rosner MR (1987) Two independent growth factor-generated signals regulate c-fos and c-myc mRNA levels in Swiss 3T3 cells. *J Biol Chem* **262**: 1442–1445

Niedel JE, Kuhn LJ and Vandenbark GR (1983) Phorbol diester receptor copurifies with protein kinase C. *Proc Natl Acad Sci USA* **80**: 36–40

Ozols RF and Young RC (1984) Chemotherapy of ovarian cancer. *Semin Oncol* **11**: 251–263

Rahmsdorf HJ and Herrlich P (1990) Regulation of gene expression by tumor promotors. *Pharmacol Ther* **48**: 157–188

Rapp UR (1991) Role of raf-1 serine/threonine protein kinase in growth factor signal transduction. *Oncogene* **6**: 495–500

Reed DJ, Babson JR, Beatty PW, Brodie AE, Ellis WW and Potter DW (1980) High-performance liquid chromatography analysis of nanomole levels of glutathione, glutathione disulfide, and related thiols and disulfides. *Anal Biochem* **106**: 55–62

Schilder RJ and Ozols RF (1992) New therapies for ovarian cancer. *Cancer Invest* **10**: 307–315

Smeal T, Binetruy B, Mercola D, Grover-Bardwick A, Heidecker G, Rapp UR and Karin M (1992) Oncoprotein-mediated signalling cascade stimulates C-Jun activity by phosphorylation of serines 63 and 73. *Mol Cell Biol* **12**: 3507–3513

 © 2000 Cancer Research Campaign
